# Identification of a Gene Encoding a New Fungal Steroid 7-Hydroxylase and Its Functional Characterization in *Pichia pastoris* Yeast

**DOI:** 10.3390/ijms242417256

**Published:** 2023-12-08

**Authors:** Vyacheslav Kollerov, Sergey Tarlachkov, Andrei Shutov, Alexey Kazantsev, Marina Donova

**Affiliations:** 1 Federal Research Center «Pushchino Center for Biological Research of the Russian Academy of Sciences», G.K. Skryabin Institute of Biochemistry and Physiology of Microorganisms, Russian Academy of Sciences, Prospekt Nauki, 5, 142290 Pushchino, Russia; sergey@tarlachkov.ru (S.T.); w___w@rambler.ru (A.S.); mv_donova@rambler.ru (M.D.); 2 Chemical Department, Moscow State University, GSP-1, Leninskiye Gori, 1, 119991 Moscow, Russia; mak@org.chem.msu.ru

**Keywords:** steroids, 7β-hydroxylation, P450-monooxygenase, *Curvularia* sp., transcriptome, recombinant *Pichia pastoris*

## Abstract

The hydroxylation of steroids in the C7β position is one of the rare reactions that allow the production of value-added precursors in the synthesis of ursodeoxycholic acid and other pharmaceuticals. Recently, we discovered this activity in the ascomycete *Curvularia* sp. VKM F-3040. In this study, the novel gene of 7-hydroxylase (P450_cur_) was identified as being heterologously expressed and functionally characterized in *Pichia pastoris*. Transcriptome data mining and differential expression analysis revealed that 12 putative genes in *Curvularia* sp. mycelia significantly increased their expression in response to dehydroepiandrosterone (DHEA). The transcriptional level of the most up-regulated cytochrome P450_cur_ gene was increased more than 300-fold. A two-gene construct with a candidate P450_cur_ gene and the gene of its natural redox partner, NADPH-cytochrome P450 reductase (CPR), which is interconnected by a T2A element, was created. Using this construct, recombinant *P. pastoris* strains co-expressing fungal P450_cur_ and CPR genes were obtained. The functional activity of the recombinant P450_cur_ was studied in vivo during the bioconversion of androstane steroids. The fungal 7-monooxygenase predominantly catalyzed the 7β-hydroxylation of androstadienedione (ADD), DHEA, and androstenediol, whereas 1-dehydrotestosterone was hydroxylated by P450_cur_ mainly at the C7-Hα position. To our knowledge, this is the first report of a recombinant yeast capable of catalyzing the 7α/β-hydroxylation of ADD and DHEA.

## 1. Introduction

The diversity and uniqueness of fungal monooxygenases capable of catalyzing the regio- and stereospecific hydroxylation of steroids make them important in the field of the microbiological synthesis of valuable hydroxysteroids, with many advantages over complicated, low-efficiency, and non-ecological chemical methods [1,2]. All currently known fungal hydroxylases involved in the hydroxylation of steroid substrates are mostly associated with enzymes of the cytochrome P450 family (CYPs), localized in the endoplasmic reticulum, and require the presence of NADPH-cytochrome P450 reductase (CPR) for electron transfer [3]. The gene expression of most fungal hydroxylases is usually activated in the presence of steroids [4,5].

Despite the ability of some mold fungi to catalyze hydroxyl group insertion into different positions of steroid molecules, the selectivity of the target reaction is often insufficient. The targeted process of hydroxylation by whole cells is accompanied by side reactions due to the presence of undesirable enzymatic activities, such as those of other hydroxylases and/or oxidoreductases (5α-reductase, 3β-hydroxysteroid dehydrogenase/isomerase, 17β-hydroxysteroid dehydrogenase, 3-ketosteroid-1(2)-dehydrogenase), as well as C-17–C-20 lyase [6,7]. This, in turn, can reduce the overall efficiency of bioconversion and complicate the recovery of the target hydroxylated steroid products.

A promising approach to solving the problem is the heterologous expression of the corresponding CYP gene in a suitable microbial host. In the initial stage, it is necessary to identify the genes involved in the target hydroxylation process in a wild-type fungal strain.

To date, reports on genes encoding specific steroid hydroxylases in filamentous fungi are very few. For example, *cyp5311b1* identified in *Absidia coerulea* AS3.65 was shown to encode 11α-hydroxylase, and its expression was induced by 16,17α-epoxyprogesterone [8]. In *Cochliobolus lunatus*, a gene encoding 11β-/14α-hydroxylase P-450lun was demonstrated to be related to the specific 14α- and 11β-hydroxylation of androstenedione and cortexolone, respectively [9,10]. A new 11α-steroid hydroxylase, CYP509C12, was identified in a *Rhizopus oryzae* strain. It was induced by progesterone, and its steroid substrate spectrum also included testosterone, 11-deoxycorticosterone, and 11-deoxycortisol, with hydroxylation occurring mainly at the 11α and 6β positions of the steroid nucleus [11]. The genes coding for three P450 enzymes (CYP5150AP2, CYP5150AP3, and CYP5150AN1) with different hydroxylase activities towards 11-deoxycortisol and testosterone were identified in the fungus *Thanatephorus cucumeris* NBRC 6298. The relevant *Pichia pastoris* recombinants were obtained, one of which (expressed *cyp5150ap2)* catalyzed 19- and 11β-hydroxylation, while the others (expressing *cyp5150ap3* and *cyp5150an1*) provided hydroxylation at the positions C7β and C2β of the steroid core, respectively [7,12]. 

During our previous wide screening of *Ascomycota* and *Zygomycota* filamentous fungi by activity towards 3-oxo steroids of the androstane series, the soil-originated ascomycete strain *Curvularia* sp. VKM F-3040 (syn. *Drechslera* sp. Ph F-34) was selected as capable of catalyzing the 7β- and 7α-hydroxylation of androstenedione (AD) and androstadienedione (ADD), activities that are quite rare in micromycetes [13]. In subsequent studies, the inducible nature of the detected 7-hydroxylase was confirmed, with the maximum inducing effect shown for dehydroepiandrostenedione (DHEA), which was also effectively hydroxylated by fungal monooxygenase at the positions C7β and C7α [14]. 

The resulting 7-hydroxylated steroids are known to act as potent anti-inflammatory and neuroprotective agents, as well as key precursors in the combined synthesis of valuable bile acids [6,15,16,17]. In the brain and other tissues, DHEA and some other steroids are prominently 7α-hydroxylated by CYP7B1, and the resulting derivatives can have serious effects on the brain and immune system [18]. In addition, 7α-OH-DHEA can affect human memory and cognitive processes and plays an important role in the treatment of autoimmune diseases [19].

It has been shown that the 7α-/7β-hydroxy-DHEA ratio can be used to differentiate Alzheimer’s disease from vascular dementia [20]. Moreover, 7-hydroxylated metabolites of androstane-type steroids, such as AD, ADD, and androstenediol, are of great interest as powerful anti-inflammatory and neuroprotective agents, diuretics, and key intermediates in the chemical and microbiological syntheses of valuable chenodeoxycholic and ursodeoxycholic acids [6,15,16,17,21].

In this regard, the *Curvularia* sp. fungal strain we discovered earlier, which has rare steroid 7β-hydroxylase activity, is of great interest. The identification of the gene encoding the enzyme responsible for 7-hydroxylation opens up great prospects for the selective synthesis of valuable 7-hydroxysteroids by using recombinant strains capable of heterologous expression of the fungal P450 monooxygenase gene. In addition, the identification of a new 7-hydroxylase gene would be a valuable contribution to the knowledge of the diversity of fungal cytochromes capable of catalyzing valuable rare reactions of steroid nucleus functionalization.

The aims of the present work were to identify the gene coding for a new steroid 7-monooxygenase in *Curvularia* sp. by whole transcriptome analysis and to perform its cloning and heterologous expression in a *Pichia pastoris* host. High-throughput sequencing of mRNAs from mycelia grown under various induction conditions, de novo transcriptome assembly, and transcriptome annotation were carried out. The gene with the maximum expression level in response to DHEA was co-expressed with its natural redox partner (CPR) in the yeast *P. pastoris* and functionally characterized.

## 2. Results

### 2.1. Induction of Steroid 7-Hydroxylase Synthesis in Curvularia *sp.* Mycelium and RNA Isolation

**Figure 1:**
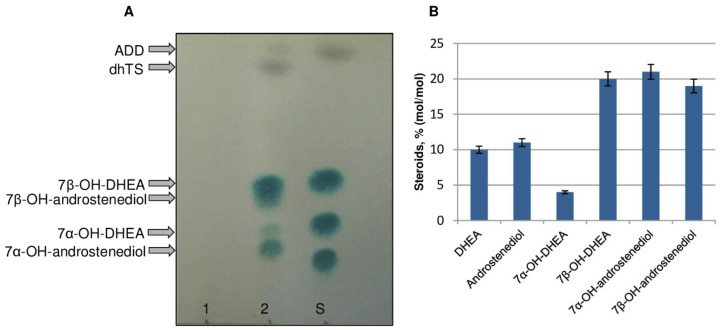
Bioconversion of DHEA by *Curvularia* sp. (**A**) TLC profile of culture broth samples (24 h, ~10 µg of steroids per spot): 1, a control non-induced mycelium; 2, a mycelium induced with DHEA (0.3 g/L) for 6 h; S, a mixture of standard steroids in the spot (top to bottom): DHEA (2 µg), 7β-OH-DHEA (2 µg), 7α-OH-DHEA (2 µg), 7α,15α-diOH-DHEA (2 µg). (**B**) HPLC data obtained for a DHEA-induced *Curvularia* sp. culture broth sample.

Cells of control non-induced and DHEA-induced *Curvularia* sp. mycelia were prepared for total RNA isolation. After a 6 h induction, culture broth samples of both variants were analyzed by TLC and HPLC to detect steroid metabolites (Figure 1). Unlike the control (non-induced) variant (Figure 1A, variant 1), a sample of the induced culture contained a mixture of steroids: non-converted DHEA, its 17β-reduced derivative androstenediol, and 7α/β-hydroxylated derivatives of DHEA and androstenediol (up to 65%, mol/mol in total) (Figure 1A (variant 2), Figure 1B), thus confirming DHEA-induced expression of the 7-hydroxylase gene in fungal cells.

### 2.2. Transcriptome Data Mining

**Figure 2:**
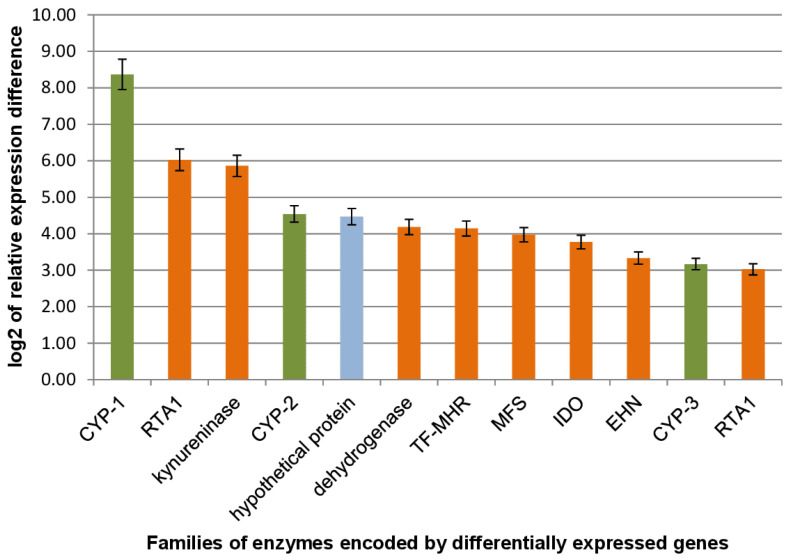
Transcript levels of the most differentially expressed genes in *Curvularia* sp. cells under DHEA induction; the hypothetical protein (blue column) does not contain any known domains.

Transcriptome data mining and differential expression analysis revealed 12 putative genes significantly up-regulated in response to DHEA. Their putative peptide sequences were subsequently used as queries to perform a BLAST search against the NCBI non-redundant protein database and to search the NCBI Conserved Domain Database for functional annotation (Figure 2). Among the genes with the highest expression level, three genes were supposed to encode cytochrome P450 (CYP) enzymes (Figure 2, green columns). Other genes were identified as putatively encoding kynureniase, NAD(P)-dependent dehydrogenase, epoxide hydrolase (EHN), transport (MFS) and transcription factor (fungal_TF_MHR) proteins, and the enzyme involved in the binding of toxic substrates (RTA1) (Figure 2, brown columns).

A sequence homology search was performed for the three candidate CYPs by BLAST and showed that CYP-1 exhibited 62.53% identity with the cytochrome P450 monooxygenase (PVI06500.1) from *Periconia macrospinosa*, CYP-2 shared 64.43% amino acid sequence identity with the hypothetical protein COCCADRAFT_40381 from *Bipolaris zeicola* 26-R-13, and CYP-3 had 65.6% homology with the cytochrome P450 monooxygenase from *Alternaria gaisen* and 73% homology with the hypothetical protein COCC4DRAFT_195011 from *Bipolaris maydis* ATCC 48331.

For further research, we selected the most up-regulated CYP-1 (hereafter referred to as P450_cur_) gene (ORF 1551 bp), which encoded a protein of 516 amino acid residues (molecular weight 58.2 kDa) and whose transcript level was induced at least 300-fold compared to the non-induced condition. Based on the NCBI conserved domain database, the P450_cur_ enzyme was ranked among CYP503A1-like proteins.

It was assumed that the P450_cur_ gene encodes a steroid 7-hydroxylase in the *Curvularia* sp. mycelium. In this regard, the next work was aimed at amplifying, cloning, and heterologously expressing the selected candidate P450_cur_ gene in *P. pastoris* with functional characterization of the recombinant P450_cur_ enzyme in vivo.

In addition, the NADPH-cytochrome P450 reductase (CPR) gene (ORF 2091 bp) encoding a 77.4 kDa protein of 696 amino acid residues, which is a natural redox partner of CYP enzymes, was identified among *Curvularia* sp. transcripts. The CPR protein was similar (97.84% identity) to the cytochrome P450 reductase 1 (ABW86977.1) from *Curvularia lunata* (protein BLAST data). Differential expression analysis revealed comparable expression levels of the CPR transcript in the control and DHEA-induced *Curvularia* sp. mycelia, thus indicating the constitutive character of the CPR enzyme.

We chose a strategy to create a two-gene construct of the P450_cur_ and CPR genes based on the pBluescriptII KS (+) and pPICZA plasmid vectors, and the *P. pastoris* GS115 yeast strain was used as a host for the heterologous co-expression of the target fungal genes.

**Figure 3:**
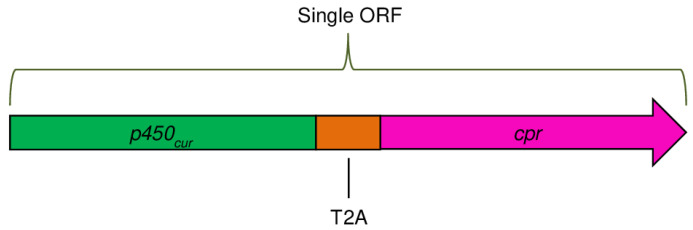
The two-gene construct with the P450_cur_ and CPR genes of the *Curvularia* sp. strain was created for cloning into the pPICZA yeast vector and heterologous co-expression in *P. pastoris*.

To synthesize the independent P-450_cur_ and CPR enzymes in recombinant yeast cells, a 63-bp sequence encoding the T2A self-cleaving peptide from the genome of the *Thosea asigna* virus was inserted between the P450_cur_ and CPR genes during the creation of the two-gene construct (Figure 3). The peptide is capable of self-cutting from the polypeptide chain at the translation stage in eukaryotic cells. It should be noted that the stop codon was removed from the P450_cur_ gene to obtain a single ORF.

### 2.3. Heterologous Expression of p450_cur_ and cpr in P. pastoris

The recombinant plasmid pPICZA-P450_cur_-CPR (see Materials and Methods for details) was linearized by digestion with PmeI and electroporated into *P. pastoris* GS115 electro-competent cells. Positive transformants were selected on YPD plates containing 100, 500, 1000, and 2000 µg/mL of the antibiotic Zeocin (see Materials and Methods for details). The corresponding empty vector pPICZA was also transformed into *P. pastoris* GS115 as a negative control.

After a 72 h incubation, the growth of positive transformants was detected on the YPD plates containing 100, 500, and 1000 µg/mL Zeocin (Figure S4B–H). No colonies were observed on the YPD plate with 2000 µg/mL Zeocin (Figure S3I) and on the control plate, where Zeocin (100 µg/mL) completely inhibited the growth of the *P. pastoris* GS115 parent strain (Figure S4A).

The positive transformant growth observed in the variants with yeast cells electroporated with the recombinant plasmid pPICZA-P450_cur_-CPR evidenced its successful integration within the 5′*AOX1* region in the corresponding region of the *P. pastoris* genome, conferring antibiotic resistance on recombinant yeast cells.

The presence of the two-gene construct with the P450_cur_ and CPR genes in the genomes of several selected positive transformants grown on a YPD medium with 100, 500, or 1000 µg/mL Zeocin was confirmed by a PCR analysis with the primer pair FPCPR/RPCPR (to amplify the CPR gene) and isolated genomic DNA or a colony lysate as a template (see Materials and Methods for details).

**Figure 4:**
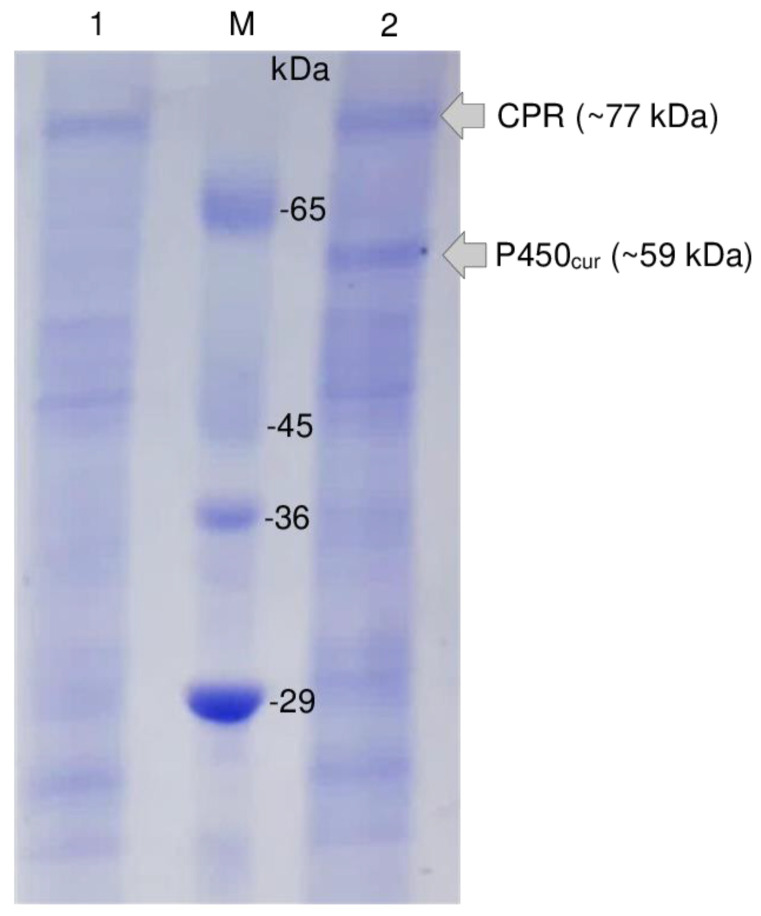
SDS-PAGE analysis of cell lysates of the control *P. pastoris* GS115-pPICZA strain (variant 1) and the recombinant *P. pastoris* GS115-pPICZA-P450_cur_-CPR strain (a positive transformant selected on the YPD medium with 1000 µg/mL Zeocin, variant 2). Cells were incubated in a BMGY medium and induced with methanol (0.5% *v*/*v*) for 24 h. M, protein molecular weight markers, kDa.

To test the heterologous expression of the target P450_cur_ and CPR genes in the recombinant *P. pastoris* GS115-pPICZA-P450_cur_-CPR strain selected on the YPD medium with 1000 µg/mL Zeocin, yeast cells were incubated in a BMGY medium and induced with methanol (0.5% *v*/*v*) for 24 h with subsequent SDS-PAGE of isolated proteins (see Materials and Methods for details). Unlike in the control variant with *P. pastoris* carrying the empty vector pPICZA (Figure 4, variant 1), a new band corresponding to a ~59 kDa protein and a more intense staining of the band corresponding to a ~77 kDa protein were detected in the variant with recombinant *P. pastoris* GS115-pPICZA-P450_cur_-CPR, thereby indicating the synthesis of the fungal P450_cur_ and CPR enzymes (Figure 4, variant 2).

### 2.4. Functional Characterization of Heterologous P450_cur_

#### 2.4.1. Bioconversion of ADD by *P. pastoris* Recombinant Strains

**Figure 5:**
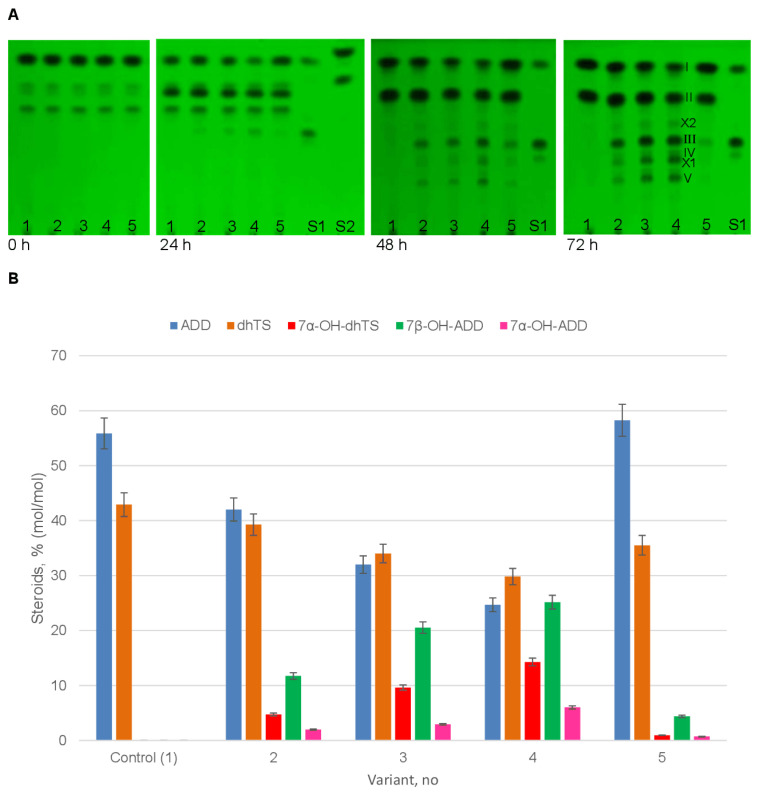
TLC profiles of culture broth samples (0–72 h) (**A**) and HPLC data (72 h) (**B**) on ADD (0.2 g/L) bioconversion by the *P. pastoris* GS115 parent strain (variant 1) and the recombinant *P. pastoris* GS115-pPICZA-P450_cur_-CPR strains, which co-expressed the P450_cur_ and CPR fungal genes and were selected on the YPD medium supplemented with 100 (variant 5), 500 (variants 2, 3), or 1000 (variant 4) µg/mL Zeocin (~10 mkg of steroids per spot). I, ADD; II, 1-dehydrotestosterone (dhTS); III, 7β-OH-ADD; IV, 7α-OH-ADD; V, 7α-OH-dhTS; S1, a mixture of standard steroids (top to bottom): ADD, 7β-OH-ADD, and 7α-OH-ADD; S2, a mixture of standard steroids (top to bottom): AD and testosterone (TS).

The biocatalytic activity towards ADD was studied in four recombinant strains selected on the YPD medium supplemented with 100 (one strain), 500 (two strains), or 1000 (one strain) µg/mL Zeocin in comparison with the control yeast strain, which carried the empty vector pPICZA (Figure 5).

No 7α/β-hydroxylated derivatives were detected in the control samples (the *P. pastoris* GS115-pPICZA recombinant strain). The only ADD metabolite was identified as 1-dehydrotestosterone (dhTS), thus evidencing the presence of an endogenous 17β-hydroxysteroid dehydrogenase (17β-HSD) in *P. pastoris* cells (Figure 5, variant 1, Figure S6).

When the recombinant *P. pastoris* GS115-pPICZA-P450_cur_-CPR strains (variants 2–5) were used for ADD bioconversion, the 17β-HSD activity of yeast cells was accompanied by 7α- and 7β-hydroxylation activities, providing for an accumulation of 7α- and 7β-hydroxylated derivatives of both the steroid substrate and its 17β-reduced intermediate. The maximum 7α/β-hydroxylase activity was observed for the recombinant strain that was selected on the YPD medium supplemented with 1000 µg/mL Zeocin (Figure 5, variant 4; Figure S6). The weakest activity was noted for the recombinant strain that was selected on the YPD medium supplemented with 100 µg/mL of the antibiotic (Figure 5, variant 5).

The major ADD hydroxylation product was identified as 7β-OH-ADD. Its maximum yield (25.15% mol/mol) was noticed in variant 4 by 72 h (Figure 5, variant 4; Figure S6). Among other metabolites, 7α-OH-ADD (6.01% (mol/mol)) and 7α-OH-dhTS (14.28% (mol/mol)) were detected (Figure 5, variant 4; Figure S6). Further incubation (96 h) did not significantly change the yield of the accumulating 7-hydroxylated metabolites. Two undefined derivatives, X1 and X2, with R_T_ 7.37 and 20.29, respectively, were detected in variant 4 by HPLC, but the structures of the metabolites were not determined due to their low amount or a concomitant formation of several metabolites (Figure S6).

#### 2.4.2. Bioconversion of DHEA by *P. pastoris* Recombinant Strains

**Figure 6:**
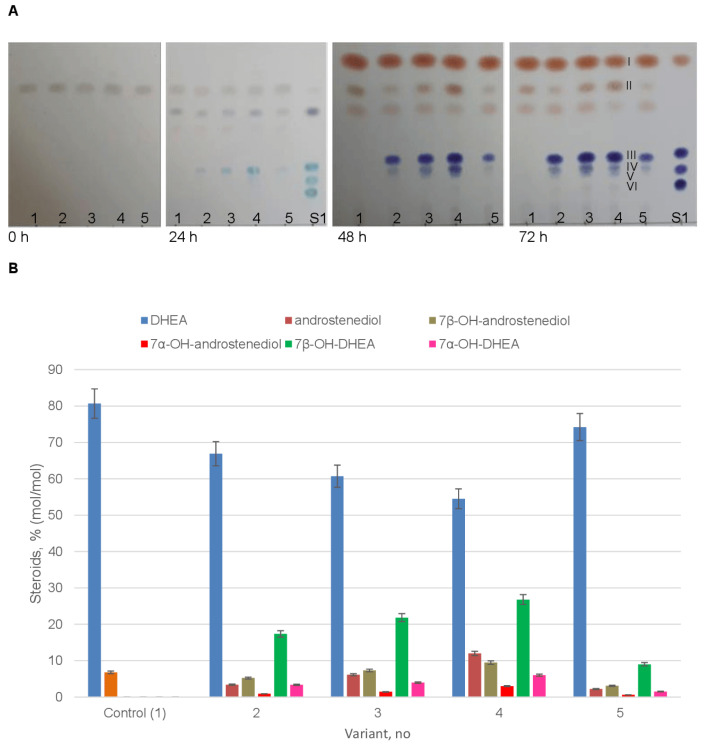
TLC profiles of culture broth samples (0–72 h) (**A**) and HPLC data (72 h) (**B**) on DHEA (0.2 g/L) bioconversion by the *P. pastoris* GS115 parent strain (variant 1) and recombinant *P. pastoris* GS115-pPICZA-P450_cur_-CPR strains, which co-expressed the P450_cur_ and CPR fungal genes and were selected on the YPD medium supplemented with 100 (variant 5), 500 (variants 2, 3), or 1000 (variant 4) µg/mL Zeocin (~10 mkg of steroids per spot). I, DHEA; II, androstenediol; III, 7β-OH-DHEA; IV, 7β-OH-androstenediol; V, 7α-OH-DHEA; VI, 7α-OH-androstenediol; S1, a mixture of standard steroids (top to bottom): DHEA, 7β-OH-DHEA, 7α-OH-DHEA, and 7α,15α-diOH-DHEA.

The biocatalytic activity towards DHEA was additionally assayed in the recombinant yeast strains. No 7-hydroxylated derivatives were detected in the control variant 1: the only product (4–6%, mol/mol) accumulated in culture broth samples (24–96 h) was identified as androstenediol, thus confirming that 17β-HSD was present in *P. pastoris* cells and catalyzed 17-keto reduction of DHEA to form androstenediol (Figure 6, variant 1).

**Figure 7:**
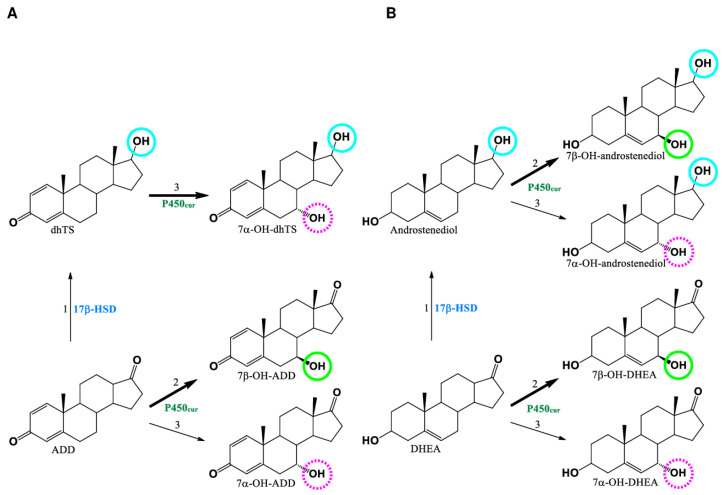
The scheme of ADD (**A**) and DHEA (**B**) transformation by the recombinant *P. pastoris* GS115-pPICZA-P450_cur_-CPR strain heterologously co-expressing the P450_cur_ and CPR genes of *Curvularia* sp.: 1, 17-keto-reduction; 2, 7β-hydroxylation; 3, 7α-hydroxylation.

At the same time, 7β-hydroxylation was the main reaction catalyzed by the recombinant *P. pastoris* GS115-pPICZA-P450_cur_-CPR strains towards DHEA, yielding 7β-OH-DHEA as the major product (Figure 6, variants 2–5). The maximum hydroxylase activity was observed in variant 4 with a 7β-hydroxylated metabolite yield of up to 26.8% (mol/mol) upon a 72 h incubation (Figure 7B). It should be noted that 7α-hydroxylation was a minor reaction and that the yield of 7α-OH-DHEA did not exceed 4–7% (mol/mol) after the whole bioconversion period (96 h). Among other steroid metabolites, androstenediol (3–12%, mol/mol) and its 7β-hydroxylated derivative 7β-androstenediol (up to 9.47%, mol/mol in variant 4) were detected, whereas only trace amounts of 7α-OH-androstenediol (<3%, mol/mol) were observed in the samples (Figure 6, variants 2–5). 

The structures of the ADD and DHEA metabolites were confirmed by mass spectrometry and ^1^H and ^13^C NMR spectroscopy analyses (see Section 4). Androstenediol and its 7α/β-hydroxylated metabolites were determined on the basis of their ^1^H and ^13^C NMR characteristic shift values, which were identical to the literature data [21].

Schemes of ADD and DHEA transformation by the recombinant *P. pastoris* GS115-pPICZA-P450_cur_-CPR strain were proposed on the basis of our results (Figure 7).

## 3. Discussion

Despite the fact that a large number of filamentous fungi of various phyla are known to catalyze the hydroxylation of steroids at various positions, a relatively small number of genes encoding steroid hydroxylases have been identified to date. For example, genes encoding 11α and 11β hydroxylases, such as CYP68J5, CYP103168, CYP5311B1, CYP5311B2, and CYP509C12, have been identified in fungi of the genera *Aspergillus, Absidia, Cochliobolus,* and *Rhizopus* [4,8,10,11,22,23]. Less information is known about the genetic control of steroid hydroxylation at other positions of the steroid core and side chain [7,24]. To the best of our knowledge, genes encoding the fungal enzyme that catalyzes the 7β-hydroxylation of androstenedione (AD) and other C19 steroids, such as ADD and DHEA, have not yet been identified.

To fill this gap, we focused in this work on the identification of the gene and the functional characterization of a new steroid 7-hydroxylase from *Curvularia* sp. strain previously chosen as a promising biocatalyst for 7β-hydroxylation of ADD.

It is known that gene expression of many fungal hydroxylases is highly induced at the transcriptional level in the presence of steroid substrates [5]. In this regard, to identify the gene encoding 7-hydroxylase in *Curvularia* sp., mycelia were grown in the presence or absence of DHEA, and the corresponding mRNA samples were obtained. The presence of clear bands of the 28S and 18S rRNAs on a gel confirmed the good quality of the *Curvularia* sp. total RNA samples (Figure S1). The samples were successfully used for mRNA isolation, sequencing, and de novo transcriptome assembly, as well as for cDNA synthesis (Figure S2). A candidate P450_cur_ gene was identified among differentially expressed transcripts in the variant with DHEA-induced *Curvularia* sp. mycelia. Its expression level increased dramatically (by more than 300-fold) in response to DHEA, suggesting a 7-hydroxylase as its product.

Most fungal steroid hydroxylases require NADPH-cytochrome P450 reductase (CPR) for electron transfer [3]. Co-expression of the CYP and CPR genes provides for the effective function of recombinant P450 enzymes [7]. For example, co-expression of the gene encoding CYP509C12 from *Rhizopus oryzae* with the gene of its native CPR partner results in a 7-fold increase in the rate of 11a-hydroxylation by recombinant yeast cells as compared to expression of CYP509C12 alone [11].

In this study, the *Curvularia* sp. gene coding for CPR was revealed among the assembled transcripts. The candidate P450_cur_ gene was co-expressed in *P. pastoris* with the gene of its native partner CPR. The yeast *P. pastoris* is well known as a suitable host for the heterologous expression of eukaryotic enzymes [25]. For instance, this yeast was successfully used to express the fungal hydroxylase genes while identifying the *Thanatephorus cucumeris* NBRC 6298 monooxygenases CYP5150AP3 and CYP5150AN1, which catalyze the 7β- and 2β-hydroxylation of 11-deoxycortisol, respectively [7]; *Absidia coerulea* AS3.65 CYP5311B1, which possesses 11α-hydroxylase activity towards 16,17α-epoxyprogesterone [8]; and *Colletotrichum lini* ST-1 CYP68JX, which has steroid C7α and C15α hydroxylase activities towards DHEA [24].

The two-gene construct with the P450_cur_ and CPR *Curvularia* sp. candidate genes interconnected by the T2A element-coding sequence was created using the pBluescriptII KS (+) plasmid vector and cloned into the *P. pastoris* expression vector pPICZA under the control of the *AOX1* promoter (Figure S3). The T2A element was selected in the present work as demonstrating the best effect in the co-expression of multiple genes at a desired ratio as compared to other known 2A elements, such as P2A or F2A [26].

The functional activity of heterologous P450_cur_ was verified in vivo by investigating the bioconversion of two steroid substrates, ADD and DHEA, by the *P. pastoris* GS115-pPICZA-P450_cur_-CPR recombinant strains (Figure 5 and Figure 6). The integration of the linearized plasmid pPICZA with the P450_cur_ and CPR genes into the yeast genome by homologous recombination (a single crossover) was confirmed in the recombinant strains.

A recombinant *P. pastoris* strain harboring the P450_cur_-CPR genes efficiently catalyzed mainly 7β-hydroxylation of ADD and DHEA to form 7β-hydroxy-ADD and 7β-hydroxy-DHEA, respectively, thus evidencing the functionality of the recombinant 7-hydroxylase. The corresponding C7α-isomers were observed in minor amounts. A control strain (harboring an empty vector) reduced the 17-carbonyl group of the C17-ketosteroid substrates due to the presence of endogenous 17β-hydroxysteroid dehydrogenase (17β-HSD) activity. This activity of the host organism resulted in the formation of 1-dehydrotestosterone (dhTS) and androstenediole, which underwent 7α- and 7α/7β-hydroxylation by the recombinant P450_cur_ to form the respective 7-hydroxylated derivatives (Figure 5 and Figure 6). Interestingly, androstenediole was mainly 7-hydroxylated in the β-position, while its 3-keto analog dhTS was transformed to a 7α-hydroxy derivative, thus evidencing the influence of the A-ring structure of a steroid (3-hydroxy-5-ene vs. 3-keto-4-ene) on the stereoposition of the hydroxyl group insertion. The stereoselectivity of hydroxylation by P450_cur_ depended also on the structure of the D-ring of the steroid core. A hydroxyl group present at C-17β in dhTS instead of a 17-keto group in ADD shifted the position of the hydroxyl group insertion by fungal 7-hydroxylase mainly towards C7-Hα. However, the same replacement of the C17-keto group in DHEA to C-17β in the androstenediol molecule did not affect the ratio of 7α/β-hydroxylated derivatives formed from the steroid substrates by P450_cur._

The detection of 7α-hydroxy-DHEA and 7α-hydroxyandrostenediol among the DHEA metabolites agrees with the hypothesis previously proposed for *Absidia coerulea* AM93, which is capable of transforming DHEA and androstenediol to a mixture of allylic 7-alcohols. It was postulated that the same enzyme is responsible for the oxidation of both C7-Hα and C7-Hβ bonds in 5-ene C19-steroids [27]. Correlations observed between the structure and geometry of the substrate molecule and the regioselectivity of hydroxylation also support the theory by Jones [28,29] that 7β-hydroxylation may occur in the normal-binding enzyme–substrate complex, while 7α-hydroxylation takes place in the reverse inverted-binding complex.

To date, the ability to catalyze the 7β-hydroxylation of androstane steroid AD has been shown only for *Bacillus megaterium* CYP106A1 and CYP106A2 enzymes, which additionally catalyze 6β- and 15β-hydroxylation, respectively [30], and a mutant of P450-BM3 (mP450-BM3), which is a well-known fatty acid hydroxylase of *B. megaterium* with a self-sufficient character of electron transfer [31]. In fungi, 7β-hydroxylase activity accompanied by the introduction of a hydroxyl group at the 6β position has been observed only for the CYP5150AP3 enzyme identified in *Thanatephorus cucumeris* NBRC 629 using 11-deoxycortisol and testosterone as substrates [7]. Noteworthily, no 6β-hydroxylated derivatives were detected among the metabolites produced by recombinant P450_cur_.

The ability to catalyze the 7α/β-hydroxylation of ADD and DHEA revealed for the newly identified *Curvularia* sp. 7-monooxygenase is of importance for the production of pharmaceuticals with neuroprotective, anti-inflammatory, and immunomodulatory effects, as has been shown for 7β-OH-DHEA [32], as well as therapeutics for the treatment of colitis and cerebral ischemia, as has been reported for 7α-hydroxyderivatives of androstane steroids [15].

## 4. Materials and Methods

### 4.1. Chemicals

Androst-4-ene-3,17-dione (AD), androsta-1,4-diene-3,17-dione (ADD), testosterone (TS), 3β-hydroxy-5-androsten-17-one (DHEA), and corn steep solids were obtained from Sigma-Aldrich (St. Louis, MO, USA). Yeast extract was purchased from Difco (Becton Drive Franklin Lakes, NJ, USA); the antibiotic Zeocin, from Thermo Fisher Scientific (Waltham, MA, USA). All other reagents were of the best purity grade and were from domestic commercial suppliers.

### 4.2. Microorganisms and Cultivation

A strain of *Curvularia* sp. VKM F-3040 was obtained from the All-Russian Collection of Microorganisms (VKM) at the Institute of Biochemistry and Physiology of Microorganisms, Russian Academy of Sciences. The growth conditions used to cultivate the first- and second-generation mycelia were as described previously [14].

The *Escherichia coli* DH5α strain was grown at 37 °C in a Luria-Bertani (LB) medium, which contained the following (g/L): tryptone, 10; yeast extract, 5; NaCl, 10; and agar, 15; pH 7.0.

The *Pichia pastoris* GS115 strain (Thermo Fisher Scientific, Waltham, MA, USA) was grown at 28 °C in a YPD medium, which contained the following (g/L): yeast extract, 10; peptone, 20; dextrose, 20 g; and agar, 15.

### 4.3. Induction of 7-Hydroxylase Activity

After 18 h of incubation, a second-generation *Curvularia* sp. mycelium culture was supplemented with 0.03% (*w*/*v*) DHEA in 2% ethanol (*v*/*v*), whereas only ethanol of the same concentration was added to a control mycelium culture. After 6 h of induction, the mycelium was collected by centrifugation (2000× *g*, 40 min) and stored at −70 °C or used immediately for total RNA isolation.

### 4.4. RNA Isolation

Total RNAs were isolated from the induced and control samples of *Curvularia* sp. mycelia using an RNeasy Mini Kit reagent (Qiagen, Redwood City, CA, USA) according to the manufacturer’s instructions. The purity and quantity of total RNAs were determined by gel electrophoresis and spectrometry with a Nanodrop 2000c instrument (Thermo Fisher Scientific, Waltham, MA, USA).

Qualitative analysis of the total RNA samples isolated from the control non-induced and DHEA-induced mycelia (two replicates for each variant) revealed two clear bands corresponding to the 28S and 18S ribosomal RNAs, evidencing the successful isolation of total RNA from the fungal cells (Figure S1). Spectrophotometric analysis of the total RNA samples confirmed their purity. The A260/280 ratio ranged from 2.11 to 2.21 (a pure RNA sample without impurities usually has an A260/280 ratio of about 2), indicating the absence of protein contamination.

### 4.5. Transcriptome Assembly, Annotation, and Gene Expression Analysis

The prediction of the coding sequences in a previous *Curvularia* sp. VKM F-3040 transcriptome assembly (accession number DDBJ/ENA/GenBank GKMF00000000) [33] was performed using TransDecoder [34]; redundant isoforms were then removed. Adapter sequences and low-quality areas found in raw reads obtained previously [33] were removed using Trimmomatic [35]. The remaining clean reads were mapped to the transcriptome assembly using Bowtie2 [36], and the mapped reads were counted using featureCounts [37]. Differential gene expression was evaluated using DESeq2 [38]. A gene was considered to significantly change in expression level if padj was <0.01 and fold change was >8.

### 4.6. cDNA Synthesis

Reverse transcription to synthesize the first cDNA strand was performed using 1 µg of the total RNA isolated from DHEA-induced *Curvularia* sp. cells and M-MLV Reverse Transcriptase in a 15 μL reaction volume according to the protocols of the manufacturer (a Mint-2 cDNA synthesis kit, Evrogen, Executive Blvd Farmingdale, NY, USA). Double-stranded cDNA (ds cDNA) was amplified using 1 µL of the first cDNA strand and 2 µL of the primer M1 (10 mM) according to the protocol of the manufacturer. Therefore, the ds cDNA samples thus obtained were enriched in full-length coding sequences and 5′- and 3′-untranslated regions. The results of ds cDNA amplification are presented in Figure S2 (looking like a smear with lengths of 0.5 to 3 kb on a gel) and confirmed the good quality of the *Curvularia* sp. first cDNA strand.

### 4.7. RT-PCR and Heterologous Expression of Candidate P450_cur_ and CPR Genes in Pichia Pastoris

The P450_cur_ and CPR candidate genes were amplified by RT-PCR using the first cDNA strand as a template; the gene-specific primer pairs FPP450_cur_/RPP450_cur_ and FPCPR/RPCPR, respectively (Table S1); and Q5 High-Fidelity DNA Polymerase (New England BioLabs, Ipswich, MA, USA). A yeast consensus sequence (6 nucleotides ACAATA preceding the start codon ATG) and a “self-cleaving” T2A element sequence were incorporated into the forward and reverse *p450_cur_* primers, respectively (Table S1). The RT-PCR conditions for *p450_cur_* amplification were as follows: initial denaturation (98 °C, 30 s); 20 cycles of denaturation (98 °C, 10 s), annealing (63 °C, 30 s), and extension (72 °C, 45 s); and final extension at 72 °C for 2 min. The RT-PCR conditions for *cpr* amplification were as follows: initial denaturation (98 °C, 30 s); 20 cycles of denaturation (98 °C, 10 s), annealing (62 °C, 30 s), and extension (72 °C, 1 min); and final extension at 72 °C for 2 min. The expected sizes were 1.6 and 2.1 kb for P450_cur_ and CPR amplicons, respectively (Figure S3A,B).

The PCR product of the P450_cur_ gene was cloned into the EcoRI and BamHI restriction sites of the pBlueScript II SK (+) vector to generate recombinant pBlue-P450_cur_. The PCR product of the CPR gene was cloned into the BamHI and NotI restriction sites of the pBlueScript II SK (+) vector to generate recombinant pBlue-CPR. The identity of the P450_cur_ and CPR genes in the recombinant plasmids was verified by sequencing. To create a two-gene construct of the P450_cur_ and CPR genes in pBlueScript II SK (+), the CPR gene was excised from the plasmid pBlue-CPR by digestion with BamHI and NotI and cloned into the corresponding restriction sites of the plasmid pBlue-P450_cur_ to generate recombinant pBlue-P450_cur_-CPR (Figure S3E). Positive *E. coli* transformants containing the recombinant plasmids were selected onan LB medium with ampicillin (100 µg/mL).

The construct of the P450_cur_ and CPR genes was excised from the plasmid pBlue-P450_cur_-CPR by digestion with EcoRI and NotI and ligated into the corresponding restriction sites of the *P. pastoris* expression vector pPICZA under the control of the methanol-induced alcohol oxidase (*AOX1)* promoter to generate the recombinant plasmid pPICZA-P450_cur_-CPR (Figure S3A). Positive transformants of *E. coli* DH5α-pPICZA-P450_cur_-CPR were selected on a Low Salt LB medium (1% tryptone, 0.5% yeast extract, 0.5% NaCl, pH 7.5) with the Zeocin antibiotic (25 μg/mL).

**Figure 8:**
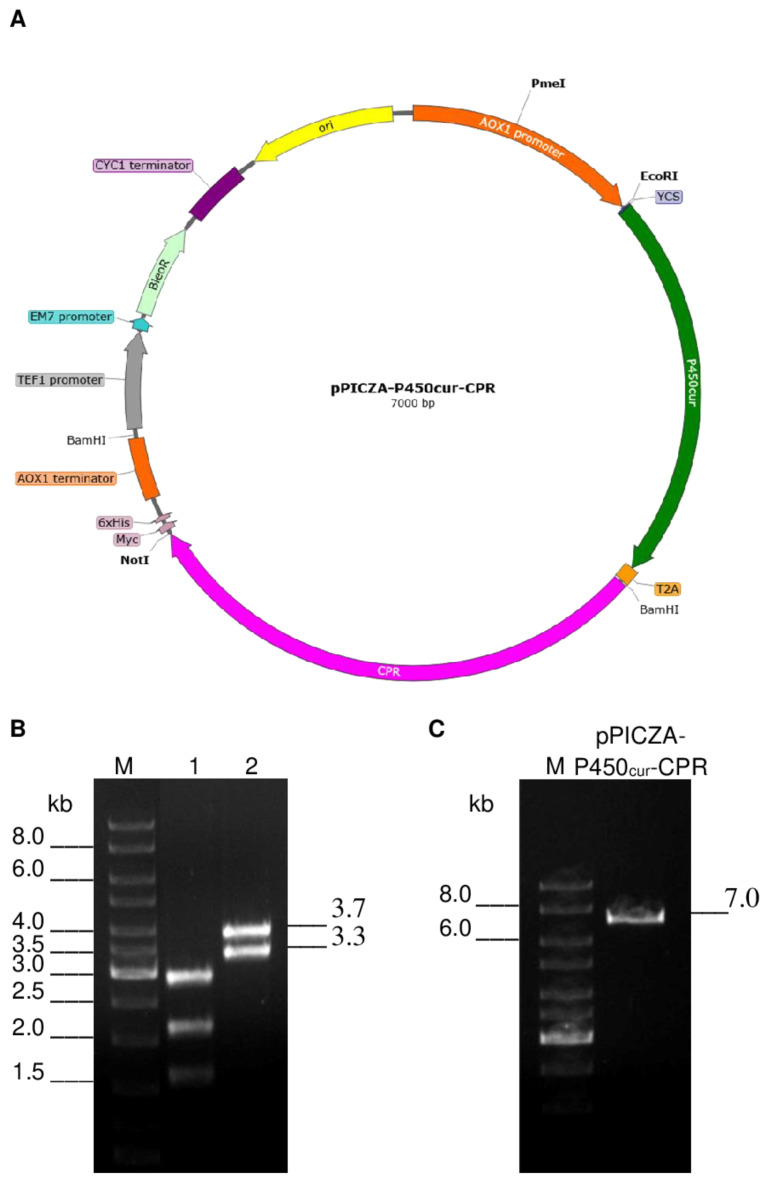
The map of the pPICZA-P450_cur_-CPR recombinant plasmid (**A**) and visualization of plasmid digestion products obtained with EcoRI, BamHI, and NotI (variant 1); EcoRI and NotI (variant 2) (**B**); or PmeI (**C**).

The presence of the two-gene construct with the P450_cur_ and CPR genes in pPICZA-P450_cur_-CPR was confirmed by digesting the recombinant plasmid with EcoRI, BamHI, and NotI. The digest showed three bands on a gel, which corresponded to the pPICZA plasmid with a deletion of a ~0.4 kb region between the EcoRI and BamHI restriction sites (upper band, ~2.9 kb), the CPR gene (middle band, ~2.1 kb), and the P450_cur_ gene (lower band, ~1.6 kb) (Figure 8B, variant 1). A digest obtained with EcoRI and NotI showed two bands on a gel, which corresponded to the pPICZA plasmid (lower band, ~3.3 kb) and the two-gene construct with the P450_cur_ and CPR genes (upper band, ~3.7 kb) (Figure 8B, variant 2). Digestion with PmeI produced one band (~7 kb), which corresponded to pPICZA (~3.3 kb) carrying the construct with the P450_cur_ and CPR genes (~3.7 kb) (Figure 8C). The identity of the P450_cur_ and CPR genes in the recombinant plasmid pPICZA-P450_cur_-CPR was verified by sequencing.

The resulting plasmid pPICZA-P450_cur_-CPR (~5 μg) was digested with the PmeI restriction enzyme to obtain a linearized form and electroporated into *P. pastoris* GS115 electro-competent cells, following the manufacturer’s protocol (MicroPulser, Bio-Rad, Hercules, CA, USA). All manipulations for preparing the *P. pastoris* electro-competent cells were performed according to the manufacturer’s protocol of an EasySelect Pichia Expression Kit (Invitrogen, Waltham, MA, USA). The ligation mixture (10–200 µL) was transferred onto YPD plates containing 100–2000 µg/mL Zeocin to select positive transformants, including those that might carry multiple copies of the target genes.

### 4.8. SDS-PAGE Analysis

Yeast cells were harvested from a total volume of 1 mL by centrifugation at 1500 g for 2 min, re-suspended in 100 µL of distilled water and 100 µL of an SDS-PAGE sample buffer, and boiled for 5 min. The cell suspension was centrifuged again, and 10 µL of the supernatant was used for 10% SDS-PAGE.

### 4.9. DNA Isolation from P. pastoris and Confirmation of Target Gene Insertion into the Yeast Genome

Total DNA was isolated from recombinant *P. pastoris* cells in accordance with the procedure described previously [39]. The presence of the P450_cur_ and CPR genes in the genomes of several selected positive transformants was confirmed by PCR analysis with the primer pair FPCPR/RPCPR (for CPR gene amplification) and isolated genomic DNA or a colony lysate as a template. In the latter case, an individual colony was injected with a sterile spout, dissolved in 20 mL of 20 mM NaOH, and boiled for 5 min. The resulting suspension (2 µL) was used for PCR in a total volume of 25 µL (Figure S5, variants 5–7). As shown in Figure S4A, a band corresponding to the amplified product of the CPR gene (~2.1 kb) was detected on a gel for all PCR reaction samples of the selected positive transformants, thus evidencing the presence of the P450_cur_ and CPR genes in the genomes of the yeast recombinants. It should be noted that the use of genomic DNA at ≥1000 ng hindered the PCR of the target gene (Figure S5, variant 1), whereas a DNA amount ranging from 10 to 20 ng was shown to be optimal (Figure S5, variants 3,4).

The insertion of the P450_cur_ and CPR genes into the genome of a single positive *P. pastoris* transformant selected on the YPD medium with 1000 µg/mL Zeocin (variant 7) was also confirmed by PCR analyses with the primer pair FPP450_cur_/RP450_cur_, which provided for amplification of the P450_cur_ gene (~1.6 kb) (Figure S5B), and FPP450_cur_/RPCPR, which allowed amplification of the construct with the P450_cur_ and CPR genes (~3.7 kb) (Figure S5C).

### 4.10. Steroid Bioconversion

First, 25 mL of a BMGY medium (g/L: yeast extract, 10; peptone, 20; YNB, 13.4; biotin, 4 × 10^−4^; glycerol, 10; 0.1 mM potassium phosphate buffer, pH 6.0) was inoculated with single colonies of the *P. pastoris* GS115 recombinant strains and incubated at 28 °C and 250 rpm for 18 h. The cells were precipitated by centrifugation (1500× *g*, 5 min), and the suspension was diluted to an OD_600_ of 1.0 with a BMMY medium containing methanol (5%, *v*/*v*) instead of glycerol. The expression of the P450_cur_ and CPR genes was maintained by adding the same dose of methanol every 24 h.

Steroid substrates (ADD or DHEA, 0.2 g/L) were added as dimethyl sulfoxide (DMSO) solutions (the final solvent concentration did not exceed 0.5%, *v*/*v*) after a 6 h induction with methanol. Bioconversion was carried out at 28 °C and 250 rpm for 72 h and monitored daily by TLC and HPLC as described below.

### 4.11. Isolation of Steroids

Steroid metabolites were isolated from the culture broth via ethyl acetate (EtOAc) extraction and fractionated by silica gel column chromatography as described previously [40]. Individual compounds were analyzed by MS and ^1^H and ^13^C NMR methods.

### 4.12. Thin-Layer Chromatography (TLC)

Cultivation broth samples (1 mL) were extracted with 2 mL of EtOAc. The extracts were applied to ALUGRAM SIL G/UV254 TLC sheets (Düren, Germany) and analyzed as described previously [14].

### 4.13. High-Performance Liquid Chromatography (HPLC)

HPLC analysis of DHEA steroid products and intermediates was performed as described earlier [14]. The retention times (R_t_) were as follows: DHEA, 8.46 min; 7α-OH-DHEA, 3.53 min; 7β-OH-DHEA, 3.38 min; androstenediol, 6.18 min; 7α-OH-androstenediol, 2.95 min; and 7β-OH-androstenediol, 2.84 min.

HPLC analysis of ADD steroid metabolites utilized a Symmetry C18 reversed-phase column, 5 µm, 4.6 mm × 250 mm (Waters, Ireland), and a Symmetry C18 guard column, 5 µm, 3.9 mm × 20 mm (Waters, Ireland). Chromatography was carried out at 50 °C with isocratic/gradient elution at a flow rate of 1 mL/min with UV detection at 254 nm. The mobile phases were as follows: A: tetrahydrofurane:acetonitrile:H_2_O:trifluoracetic acid (10:10:80:0.02, *v*/*v*/*v/v*); B: acetonitrile:trifluoracetic acid (100:0.02, *v*/*v*). Elution was carried out according to the following scheme: 0–14 min, B: 0%; 14–28 min, B: 0–60%. The retention times (Rt) were as follows: ADD, 23.68 min; dhTS, 23.97 min; 7α-OH-ADD, 9.51 min; 7β-OH-ADD, 9.2 min; and 7α-OH-dhTS, 8.38 min.

### 4.14. Mass Spectrometry (MS), ^1^H- and ^13^C-NMR Spectroscopy

MS spectra were recorded with a Bruker Maxis Impact spectrometer.

^1^H- and ^13^C-NMR spectra were recorded at 400 and 100.6 MHz, respectively, with a Bruker Avance 400 spectrometer. Chemical shifts were measured relative to a solvent signal. Only characteristic signals in ^1^H-NMR of steroids are given.

Spectral data of DHEA and ADD steroid derivatives formed by the *P. pastoris* GS115-pPICZA-P450_cur_-CPR recombinant strains are as follows:

3β,7α-Dihydroxyandrost-5-en-17-one (7α-hydroxydehydroepiandrosterone, 7α-OH-DHEA) (M 304): ^1^H-NMR (CDCl_3_) δ: 5.64 (dd, J = 1.6, 5.2 Hz, 1H, 6-H), 3.98 (br. t, J = 3.8 Hz, 1H, 7β-H), 3.57 (tt, J = 4.7, 11.2 Hz, 1H, 3α-H), 2.51–1.08 (m, 17H), 1.02 (s, 3H, 19-CH3), 0.89 (s, 3H, 18-CH3). ^13^C-NMR (CDCl_3_) δ: 221.2 (C-17), 146.4 (C-5), 123.5 (C-6), 71.1 (C-3), 64.2 (C-7), 47.1, 44.9, 42.5, 41.9, 37.5, 37.1, 36.9, 35.8, 31.2, 31.0, 21.9, 20.0, 18.2, 13.2.

3β,7β-Dihydroxyandrost-5-en-17-one (7β-hydroxydehydroepiandrosterone, 7β-OH-DHEA) (M 304): ^1^H-NMR (CDCl_3_) δ: 5.32 (s, 1H, 6-H), 3.96 (m, 1H, 7α-H), 3.55 (m, 1H, 3α-H), 2.57–1.13 (m, 17H), 1.08 (s, 3H, 19-CH_3_), 0.90 (s, 3H, 18-CH_3_). ^13^C-NMR (CDCl_3_) δ: 221.2 (C-17), 143.6 (C-5), 125.5 (C-6), 72.8 (C-7). 71.2 (C-3), 51.1, 48.2, 47.7, 41.6, 40.4, 36.8, 36.6, 35.9, 31.4, 31.2, 24.2, 20.3, 19.1, 13.6.

Spectral data of 7α-hydroxyandrosta-1,4-diene-3,17-dione (7α-OH-ADD), 7β-hydroxyandrosta-1,4-diene-3,17-dione (7β-OH-ADD), 1,4-androstadien-17β-ol-3-one (1-dehydrotestosterone, dhTS), and 7α-hydroxyandrosta-1,4-diene-17β-ol-3-one (7α-hydroxydehydrotestosterone, 7α-OH-dhTS) were identical to those obtained previously for ADD steroid metabolites formed by the *Curvularia* sp. VKM F-3040 strain [21].

### 4.15. Quantification of Bioconversion Steroid Metabolites

The conversion rate (CR) was calculated using the following formula:

(1) $$CR=\frac{\left(mP/MP\right)}{\left(mS/MS\right)}\times 100\%,$$

where mP and mS are the weights of the steroid product and substrate, respectively, and MS and MP are their respective molar weights.

### 4.16. Accession Numbers

The nucleotide sequences of the P450_cur_ and CPR genes were deposited in the GenBank database under the accession numbers OQ632931 and OQ632932, respectively.

### 4.17. Statistical Analysis

Microsoft Excel 2010 was used to process the data. All experiments were performed in triplicate, and each value presented is the mean of three independent experiments. Standard deviations are displayed as errors in the graphs.

## 5. Conclusions

The gene of the novel steroid 7-monooxygenase P450_cur_ from a *Curvularia* sp. fungal strain was identified and functionally characterized via transcriptome analysis and gene cloning and heterologous expression in *P. pastoris*. Recombinant yeast cells heterologously expressing the fungal 7-hydroxylase gene efficiently catalyzed the 7β-hydroxylation of ADD and DHEA. The structures of steroid metabolites were confirmed by HPLC, mass spectrometry, and ^1^H and ^13^C NMR spectroscopy analyses. The results expand the knowledge on the diversity of P450 hydroxylases in lower eukaryotes and open up the prospects for selective biotechnological production of valuable 7-hydroxysteroids.

## Data Availability

The data that support the findings of this study are available from the corresponding author, V.K., upon reasonable request.

## Supplementary Materials

The following supporting information can be downloaded at: https://www.mdpi.com/article/10.3390/ijms242417256/s1.
