# Supplementary material for: Identification of a Gene Encoding a New Fungal Steroid 7-Hydroxylase and Its Functional Characterization in Pichia pastoris Yeast
Source: Int J Mol Sci. 2023 Dec 8;24(24):17256. doi: 10.3390/ijms242417256 (PMC10744122; doi:10.3390/ijms242417256)
Supplement: Supplementary file 1 [file ijms-24-17256-s001.zip › ijms-2717216-supplementary.pdf]

Identification of a Gene Encoding a New Fungal Steroid 7-Hydroxylase and Its Functional Characterization in *Pichia pastoris* Yeast

Vyacheslav Kollerov [1,*], Sergey Tarlachkov [1], Andrei Shutov [1], Alexey Kazantsev [2] and Marina Donova [1]

[1] Federal Research Center «Pushchino Center for Biological Research of the Russian Academy of Sciences», G.K. Skryabin Institute of Biochemistry and Physiology of Microorganisms, Russian Academy of Sciences, Prospekt Nauki, 5, 142290 Pushchino, Russia
[2] Chemical Department, Moscow State University, GSP-1, Leninskiye Gori, 1, 119991 Moscow, Russia
* Correspondence: svkollerov@rambler.ru; Tel.: +7-4967-318584; Fax: +7-495-9563370

Supplementary Table S1. List of oligonucleotide primers used for $P450_{cur}$ and CPR genes amplification

Gene	Forward primer (FP) (5`-3`)*	Reverse primer (RP) (5`-3`)
$p450_{cur}$	GAGACTGAATTCACAATAATGTCTACAGCCAGCATGG	GGATCCTGGGCCGGGATTTTCCTCCACGTCCCCGCATGTTAGAAGACTTCCCCTGCCCTCGCCGGAGCCACTAAATTCCAGTGCCTCC
cpr	GAGACTGGATCCATGGCACAACTCGATACGCTCG	GAGACTGCGGCCGCTCATGACCAGACATCTTCC

* Restriction sites are underlined in primers.

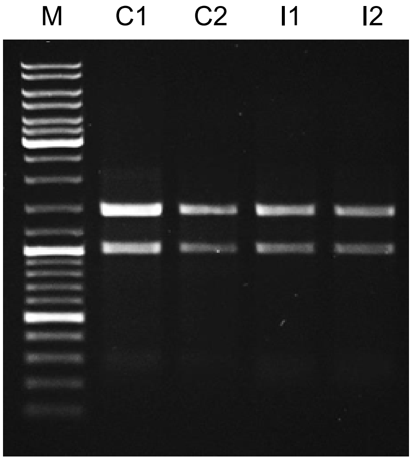

Supplementary Figure S1. Visualization of total RNA isolated from the control, non-induced (C1-C2) and DHEA-induced (I1-I2) *Curvularia* sp. mycelia; M - DNA ladder mix (0.4 μg); 1.2% (w/v) agarose/EtBr gel electrophoresis run in 1X TAE buffer at 120 V for 40 min.

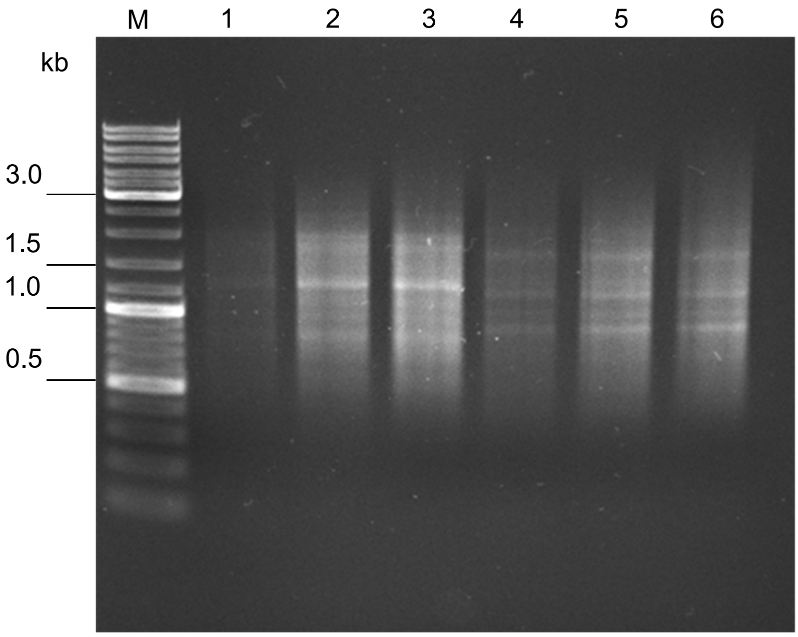

Supplementary Figure S2. Gel profile of ds cDNA synthesized from 1 μg of total RNA isolated from DHEA-induced *Curvularia* sp. (variants 1-3) and 1 μg of the control human brain total RNA (variants 4-6) following the Mint-2 cDNA synthesis kit protocol. Lanes 1, 4 – PCR product after 15 cycles; lanes 2, 5 – after 18 cycles; lanes 3, 6 – after 21 cycles; M - DNA ladder (0.5 μg).

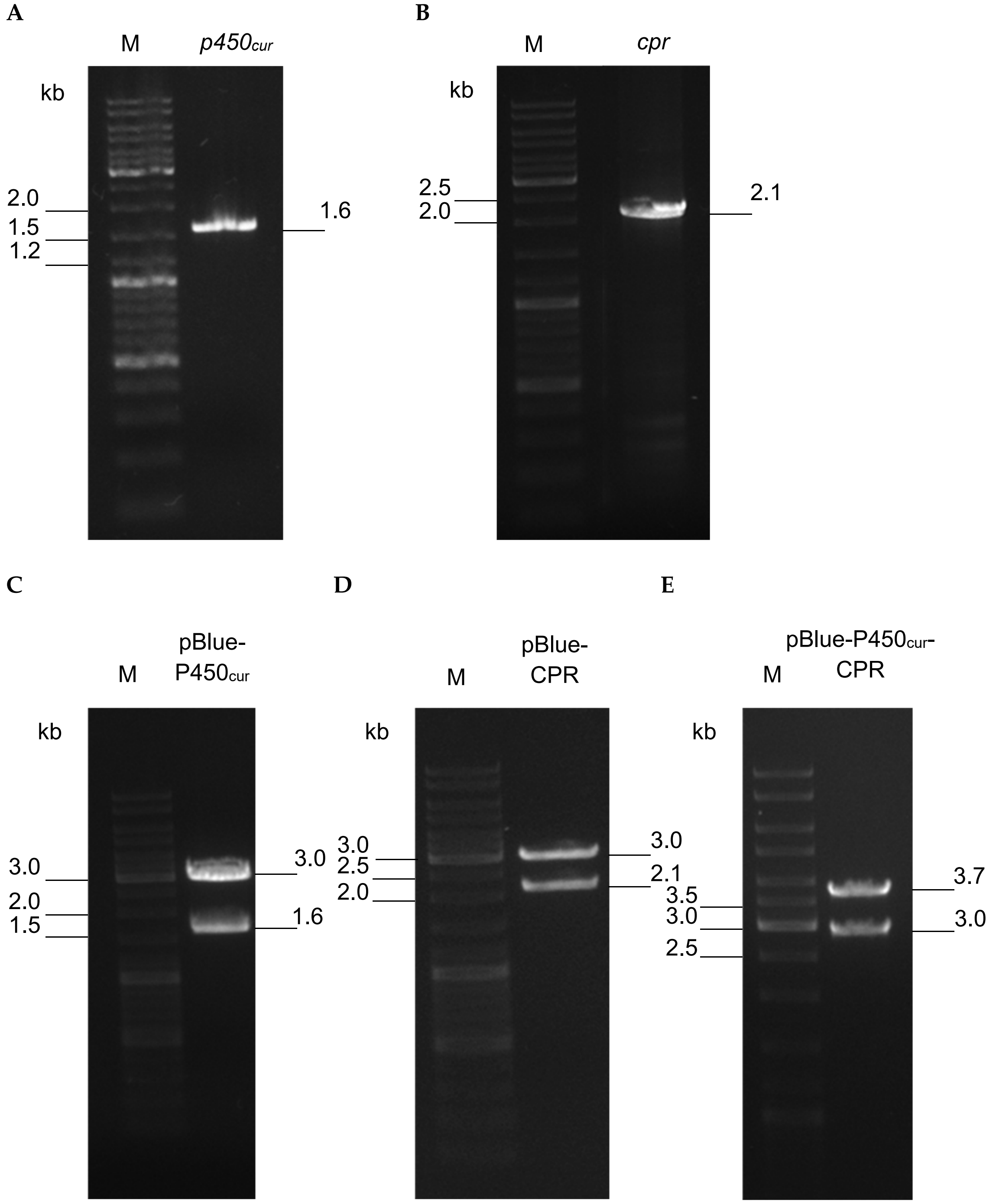

Supplementary Figure S3. Visualization of the $P450_{cur}$ (**A**) and CPR (**B**) amplified products and the recombinant plasmids pBlue-$P450_{cur}$ digested with *Eco*RI and *Bam*HI (**C**), pBlue-CPR digested with *Bam*HI and *Not*I (**D**) and pBlue-$P450_{cur}$-CPR digested with *Eco*RI and *Not*I (**E**); M - DNA ladder (0.5 μg).

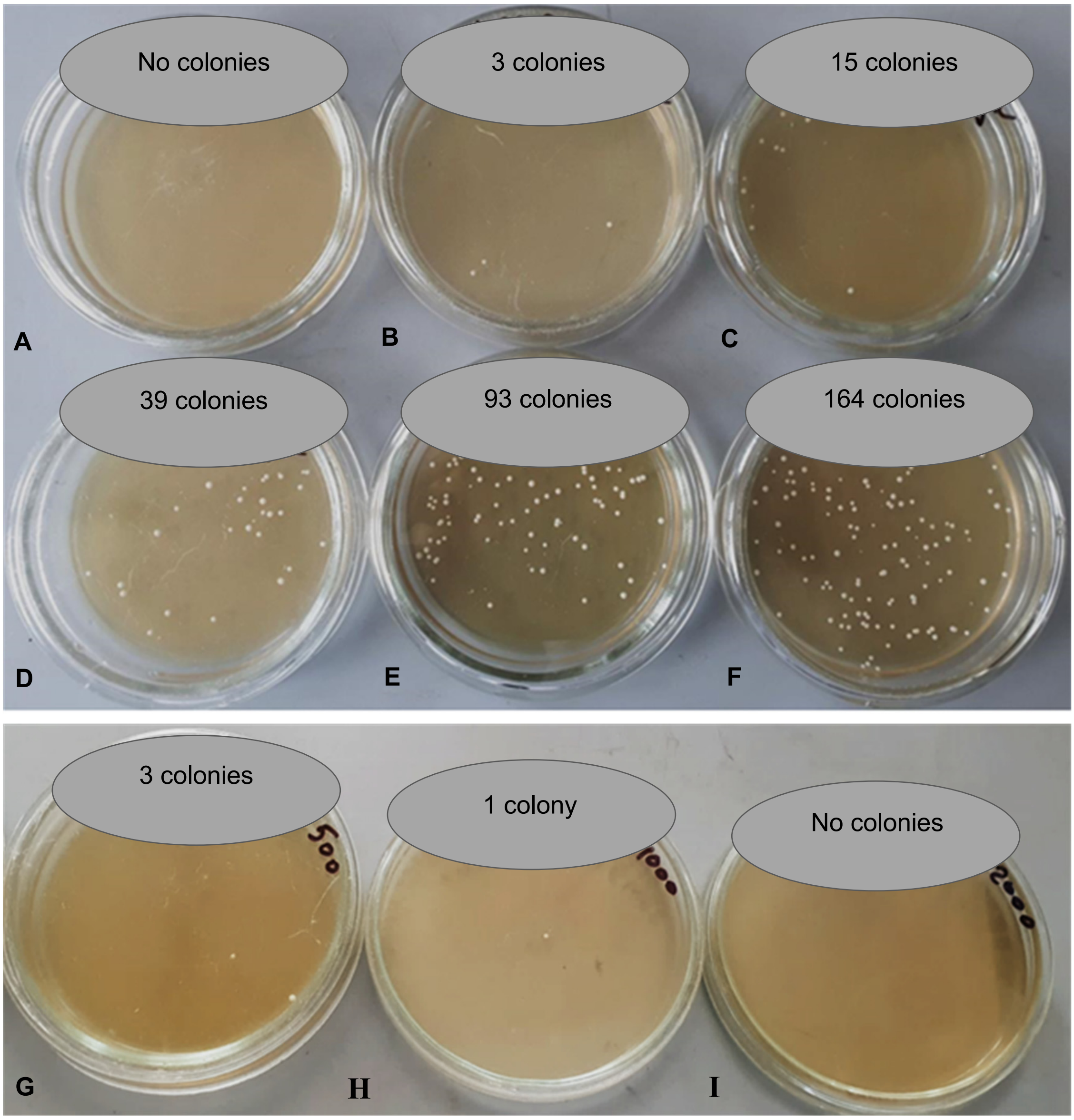

Supplementary Figure S4. The growth of *P. pastoris* GS115 parent strain (**A**) and positive tranformants electroporated by recombinant linearized plasmid pPICZA-$P450_{cur}$-CPR (**B** – **I**) on YPD plates supplemented with 100 (**A** – **F**), 500 (**G**), 1000 (**H**) and 2000 μg/mL (**I**) antibiotic Zeocin. The suspension of 100 (**A**, **E**, **G** – **I**), 10 (**B**); 25 (**C**); 50 (**D**) or 200 μL (**F**) yeast cells was transferred to YPD plate and incubated at 28^0C for 72 h.

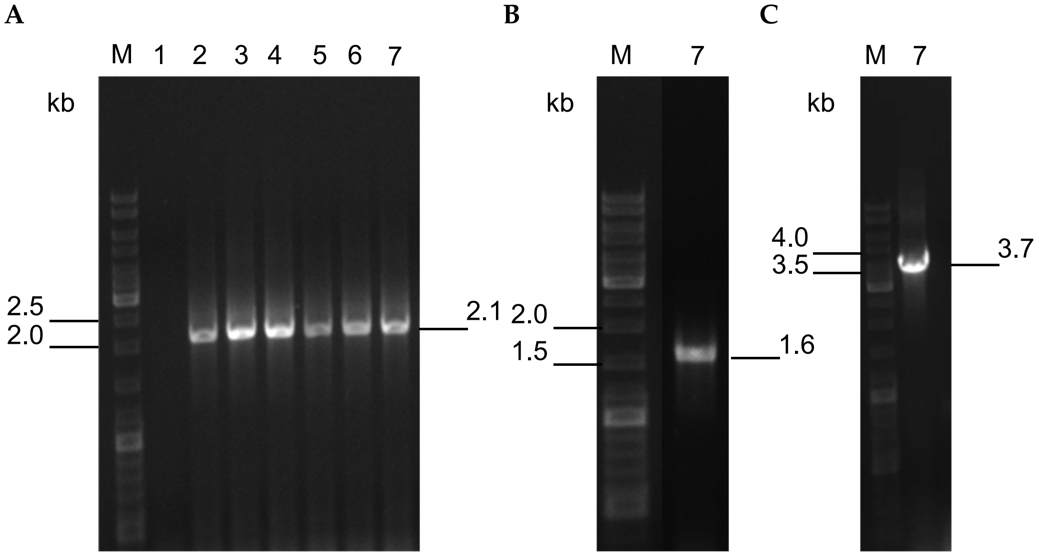

Supplementary Figure S5. Visualization of amplified products of PCR reaction (35 cycles) with the primer pair FPCPR/RPCPR (for CPR gene amplification) (**A**), FPCYP/RPCYP (for P450$_{cur}$ gene amplification) (**B**) and FPCYP/RPCPR (for two-gene construct of P450$_{cur}$ and CPR genes amplification) (**C**) of selected positive transformants grown on YPD medium with 100 (variants 1-5), 500 (variant 6) or 1000 μg/mL Zeocin (variant 7). Genomic DNA at concentration of 1000 (variant 1), 100 (variant 2), 20 (variant 3) or 10 ng (variant 4) or colony lysate (variants 5-7) was used as a template for PCR. M - DNA ladder (0.5 μg); 0.8% (w/v) agarose/EtBr gel electrophoresis run in 1X TAE buffer at 120 V for 40 min.

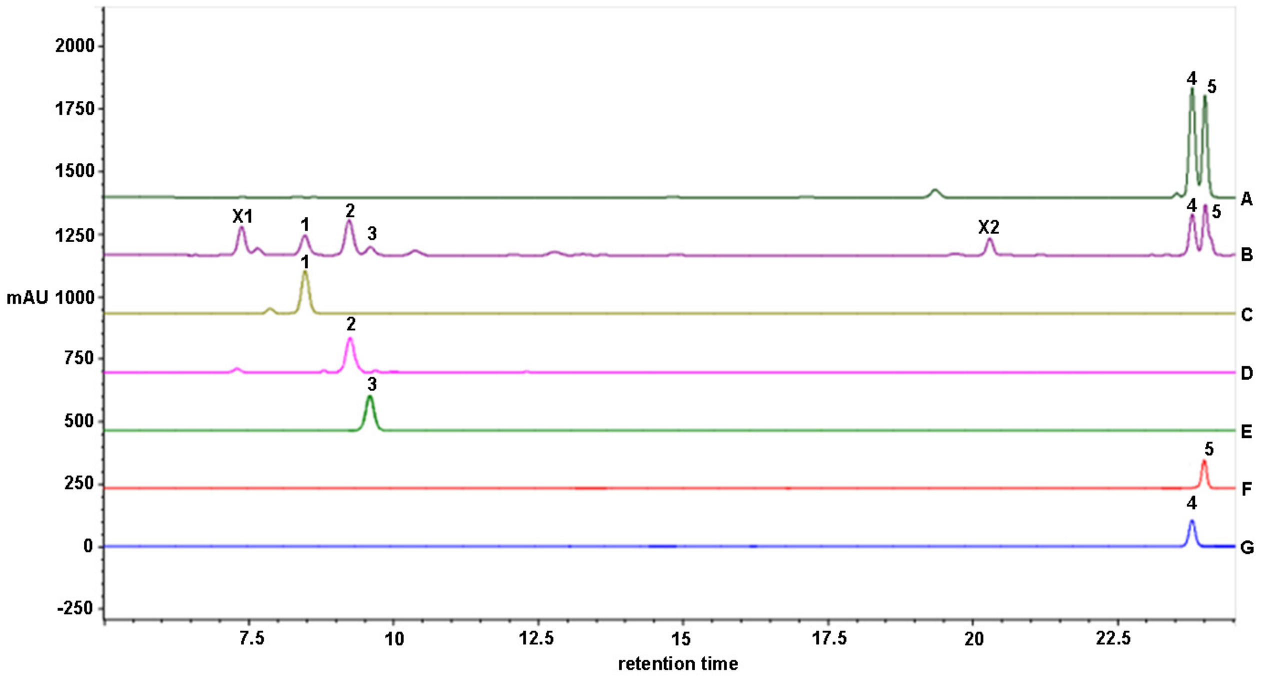

Supplementary Figure S6. HPLC chromatograms of ADD bioconversion samples (72 h): **A**, *P. pastoris* GS115-pPICZA recombinant strain carrying empty vector (control variant); **B**, *P. pastoris* GS115-pPICZA-P450$_{cur}$-CPR recombinant strain co-expressing P450$_{cur}$ and CPR genes; **C**, standard reference of 7α-OH-dhTS (1); **D**, 7β-OH-ADD (2); **E**, 7α-OH-ADD (3); **F**, ADD (4); **G**, dhTS (5).
